# Ethnobotanical indicator values of Non-Timber Forest Products from the Djoumouna peri-urban forest in Brazzaville, Republic of Congo

**DOI:** 10.1016/j.heliyon.2021.e06579

**Published:** 2021-03-27

**Authors:** V. Kimpouni, J.D.D. Nzila, N. Watha-Ndoudy, M.I. Madzella-Mbiemo, S. Yallo Mouhamed, J.-P. Kampe

**Affiliations:** a École Normale Supérieure (ENS), Université Marien Ngouabi, BP 237 Brazzaville, Congo; b Institut National de Recherche Forestière (IRF), BP 177 Brazzaville, Congo; c Faculté des Sciences et Technique (FST), Université Marien Ngouabi, BP 69 Brazzaville, Congo

**Keywords:** Brazzaville (Congo), Djoumouna peri-urban forest, NTFPs, Ethnobotanical indicator, Biodiversity

## Abstract

A study of the relationship between man and his environment was carried out in Brazzaville, Republic of Congo in the peri-urban forest of Djoumouna. Socio-economic and ethnobotanical surveys conducted among the human populations in riparian areas were supported by direct field observations and a review of the literature. Data were collected from a 2 km zone of influence around the Djoumouna forest. The group of survey participants, organized into four age groups, included all socio-professional categories and was made up of 143 heads of household. The survey participants were of both genders and ranged in age from 15 to over 45 years old. Ethnobotanical indicators were used as data analysis tools, specifically, ethnobotanical use-value, survey participant consensus factor, and level of fidelity. This study identified 13 plant species and more than 14 animal taxa as Non-Timber Forest Products (NTFPs) of animal and plant origin. Most of the taxa listed are used in traditional foods and/or phytotherapy. The low values of some ethnobotanical indicators show that these NTFPs, which are not highly sought after and valued by the population, are also rare in this peri-urban ecosystem. This observation is also valid when considering the involvement of survey participants by age group. However, the survey participant consensus factor reflects a unanimity of traditional exploitation of these NTFPs within the society. The analysis of the ethnobotanical data clearly show a difference in the level of exploitation of NTFPs between genders with men having more interest in finding and using NTFPs. Finally, the study indicates (i) gender specialization is associated with NTFP activities and exploitation, and (ii) a progressive erosion of traditional knowledge is occurring between age groups.

## 1 Introduction

The economic, social and ecological importance of Congolese forests is no longer in doubt, as they constitute an essential lever for economic emergence and development in the Republic of Congo (FAO, 2006). Forests provide society with a set of ecosystem goods and services of a national and regional scope. Ecosystem goods and services refer to all the benefits that humans derive from the functioning of ecosystems (Barnaud et al., 2011). Provisioning (harvesting) services include products such as timber, fuelwood, service wood, food, game, drinking water and medicines (Shackleton et al., 2015). Regulatory services include climate normalization, urban heat island control, carbon storage, air pollution removal, watershed-level water regulation, soil protection and flood control (Dobbs et al., 2011). Socio-cultural services relate to natural heritage, recreation, aesthetics, ecotourism and the cultural values of forests (Dobbs et al., 2011).

Urban and peri-urban forests play an effective role in the process of biodiversity conservation by providing ecological niches for many plant and animal species (Alvey, 2006). For example, hundreds of threatened native species currently depend on urban habitats in Australia (Ives et al., 2016). These forests also enhance cultural diversity by increasing the resilience of cities to environmental shocks and stressors (Colding and Barthel, 2013).

In the Congo, the intra- and peri-urban forests of the city of Brazzaville provide a wide range of goods and services, both tangible and intangible, to society. Of these, the most valued are associated with harvesting services. The materiality of the supply service is provided through Non-Ligneous Forest Products (NTFPs). Under the term, NTFPs are distinguished plant taxa (medicinal, aromatic and food plants, service wood, and energy wood) and animal taxa (bushmeat and other game products, birds, etc.).

Notwithstanding this premium of services forests provide to urban populations, intra- and peri-urban forests are subject to anthropogenic pressures of various kinds. For reasons, notably of anarchic urbanization, the city of Brazzaville, which had five intra-urban massifs in the 1970s, has seen four of them disappear, without their biodiversity being revealed (Makany, 1976; Kimpouni et al., 2013, 2014). Currently, the Patte d’Oie forest is the only natural intra-urban forest ecosystem in the city, despite the forest's high level of anthropization and the loss of more than 70% of the original forest area (Mbou, 2009; Gakosso, 2009; Ikama Openga, 2013; Kimpouni et al., 2013). Continually exposed to strong anthropogenic pressures, these ecosystems are experiencing a loss of biodiversity that affects ecosystem goods and services (Tiokeng et al., 2015). The peri-urban forest of Djoumouna is not on the margin of this degradation. The forest here is faced with several constraints, including the cutting of fuelwood, timber, the removal of various organs (bark, roots, leaves, fruits) of medicinal and food plants, and fires resulting from slash-and-burn agriculture, which affect and induce biodiversity erosion (α, β, γ) in all compartments of this forest ecosystem. In addition to these parameters, the low rate of natural regeneration of many species and the slow growth of the main forest species seriously affect the development of these ecosystems (Massamba-Makanda, 2017).

Because the capacity of the forest to supply society with NTFPs has been undermined by the level of advanced anthropogenic degradation, it has become more than urgent to assess the status of this category of forest products in the Djoumouna peri-urban forest. Among all the services, those relating to supply are by far the most sought after by local residents. Because NTFPs constitute a cardinal value in the daily satisfaction of the communities' needs, they can generate arguments related to the conservation and/or restoration of these forest areas (Peh et al., 2014). This study, which is a contribution to a better understanding of the roles and functions fulfilled by intra- and peri-urban forests, is based on (i) an inventory of NTFPs provided by the managers of Djoumouna Forest, (ii) the identification of zones of influence and (iii) the most dependent social strata.

## 2 Materials and methods

### 2.1 Presentation of the study area

**Figure 1:**
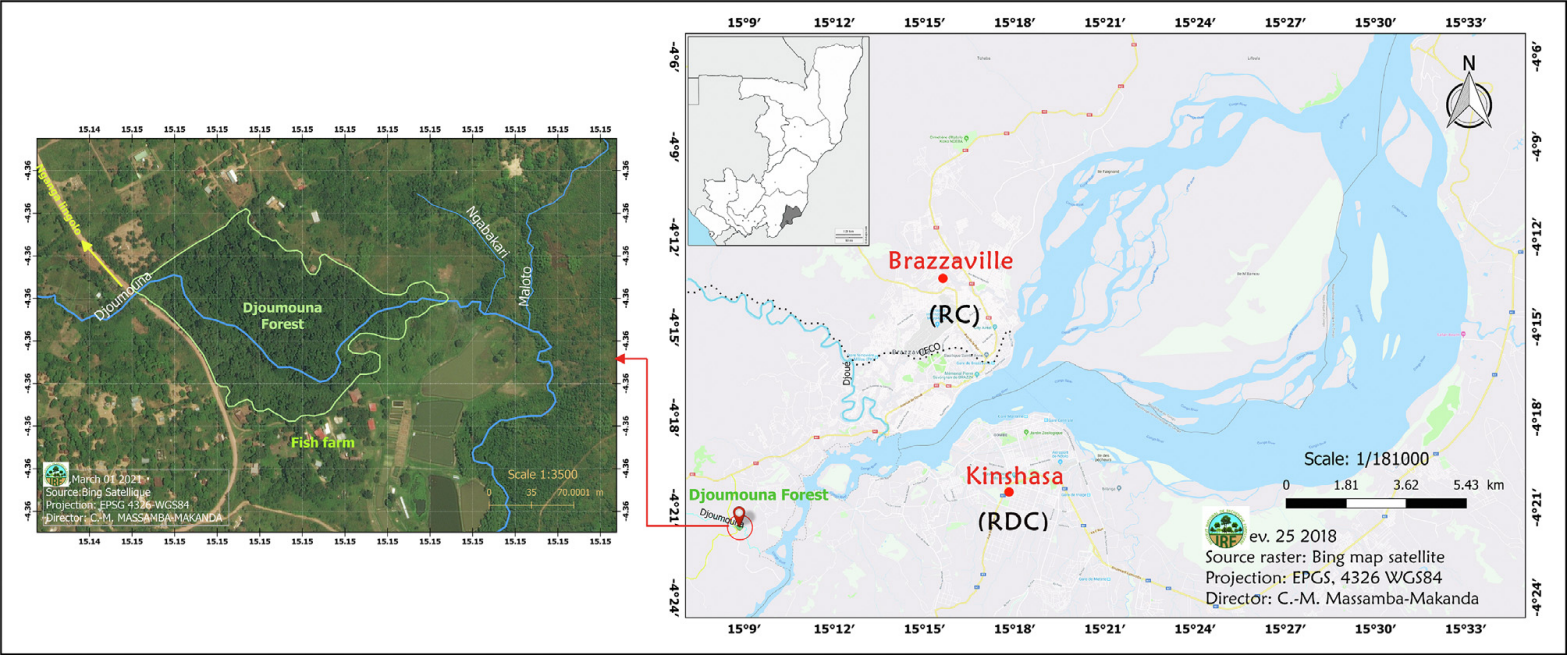
Location of the study area.

The Djoumouna Forest (−04°22′ to −04°35′S and 15°09′ to 15°15′E) is located 24 km southwest of Brazzaville (Figure 1). The Djoumouna River, a small permanent tributary of the Congo River, waters this riparian belt, which currently covers 3.5 ha. This forest is bounded to the northwest by Nganga Lingolo-Linzolo road, to the south by the Djoumouna River, and to the east by the Maloto River (Massamba-Makanda, 2017).

**Figure 2:**
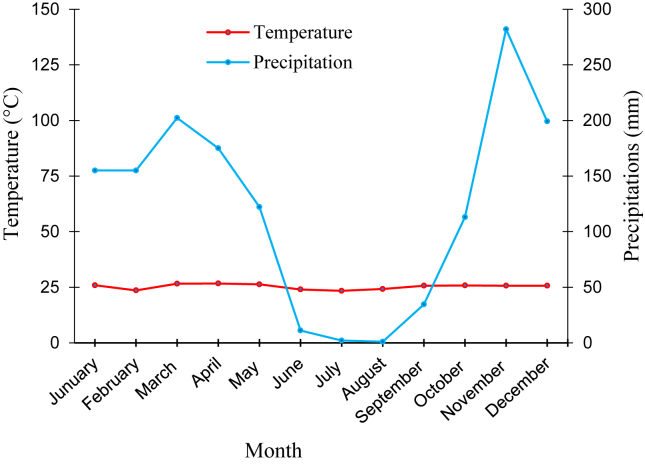
Ombrothermal curve for Brazzaville from 2003 to 2017.

This forest formation evolved under a Bas-Congolese type climate (Descoings, 1969; Samba-Kimbata, 1978), with an average annual temperature of about 25 °C, a low annual thermal amplitude from 5 to 6 °C (Figure 2), and average annual rainfall from 1200 to 1400 mm (Samba, 2014). The rains that punctuate the alternation of the seasons begin very weakly at the end of September and fall from October to May, with a very marked slowdown from January to February. The hottest and wettest months are usually March, April and November. In addition, the months of June to September are the driest, while July and August are the coolest (Samba-Kimbata, 1978; Samba-Kimbata and Mpounza, 2001).

**Figure 3:**
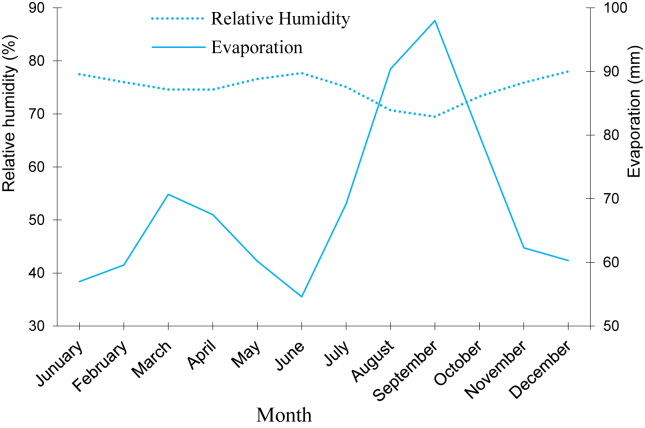
Relative humidity and evaporation curves for Brazzaville from 2003 to 2017.

Relative atmospheric humidity, always above 70%, is vital for the flora, especially in the dry season. The annual hygrometric amplitude is low and the daily average varies from 33% in the rainy season to 46% in the dry season (Samba-Kimbata, 1978; Samba, 2014). Evaporation varies in the opposite direction of atmospheric humidity (Figure 3) and presents a relative minimum in June and an absolute maximum in August and September (Samba, 2014).

The average monthly insolation, from 2003 to 2017, was between 133 and 182 h/month. The insolation peaks from March to May (181–177 Kwh/m^2^) and reaches a minimum in June (134 Kwh/m^2^). The annual average varies from 1100 to 1800 h (Massamba-Makanda, 2017; Kimpouni et al., 2018a, 2018b, [Bibr bib30][Bibr bib29]).

Winds are generally light (1.5 m/s) and are from the west, southwest and northwest (Koechlin, 1961; Samba, 2014). Exceptionally, violent winds of short duration have been observed, corresponding to the passage of tornadoes, particularly at the time of the equinoxes (Vennetier, 1977).

### 2.2 Physical setting

#### 2.2.1 Soils

The soils under the Djoumouna forest belong to the ferrallitic and hydromorphic soil classes (Denis, 1974). These soils, which are derived from predominantly siliceous rocks, have an acidic pH and lack a sufficient humus complex, resulting in very low nutrient fixation. As a result, these soils are poor (Desthieux, 1993; Alvarez et al., 1995).

Ferrallitic soils have a generally thick upper level, then a medium level of coarse elements, often indigenous (quartz sandstone gravel and pebbles, for example), and finally a lower level, known as an alteration, of mauve color with wine lees, very quartzy with numerous anastomosing whitish veins. These soils belong to the subclass of highly desaturated ferrallitic soils, with exchangeable bases (Ca, Mg, K, Na) in very low concentration in the B horizon (of the order of 1 meq/100 g) and with a saturation rate of the absorbent complex of less than 20% (Schwartz, 1987).

Hydromorphic soils have characteristics that are a result of an evolution dominated by the effects of an excess of water following a temporary or permanent waterlogging, either deep or overall, caused by the presence or rising of a water table. These soils have an organic matter content of around 10% and a relatively high C/N ratio (from 20 to 40). Thanks to the organic matter content, the exchange capacity is high but most often the rate of saturation in bases is low. Free iron levels are also typically low (Schwartz, 1987).

#### 2.2.2 Flora and vegetation

The Djoumouna forest belongs phytogeographically to the District of Cataractes, bordered by the Districts of Niari and Léfini. This mesophilic and tropophilic forest supports a flora based on species such as *Millettia laurentii*, *Pentaclethra eetveldeana*, *Staudtia kamerunensis*, *Petersianthus macrocarpus*, *Macaranga monandra* and *Trichilia monadelpha* (Miabangana, 1998; Massamba-Makanda, 2017). They are distributed based on an edaphic gradient. On clayey-sandy soils on dry land, forest facies with *Pentaclethra macrophylla* have evolved, relayed by riparian formations with *Gilbertiodendron dewevrei* on schistosandstone soils. On sandy soils, facies with *Pentaclethra eetveldeana*, *Millettia laurentii* and *Albizia zygia* develop, as well as secondary formations with *Triplochiton scleroxylon*, *Terminalia superba* and *Musanga cecropioides*; and finally, forests dominated by with *Pentadesma butyracea* and *Dialium corbisieri* occur (Miabangana et al., 2016; Massamba-Makanda, 2017). Anthropic degradation induces secondary formations, of which various heliophilic pioneer species and *Elaeis guineensis* are characteristic.

### 2.3 Study methods

The present study is based on socio-economic and ethnobotanical surveys conducted from 06 to 25 September 2018 along with direct field observations and a review of the literature. Data from the literature made it possible to take stock of biodiversity management in the urban and peri-urban woody formations in Brazzaville (Vennetier, 1966; Makany, 1976; Trouillet et al., 1982; Kimpouni et al., 2013, 2014, 2020).

Biodiversity data were collected by using and analyzing questionnaires. However, the taxa selected were those that have been materialized in the field. The identification of plant taxa was carried out simultaneously at the Botany Laboratory of the Higher Normal School of Marien Ngouabi University and at the National Herbarium (IEC) of the Congo located at the National Research Institute for Natural Sciences (IRSEN). As for the animal samples, identifications were certified at researchers at the animal biology laboratories of the Higher Normal School of Marien Ngouabi University and the National Research Institute for Natural Sciences (IRSEN).

#### 2.3.1 Study area zoning

**Figure 4:**
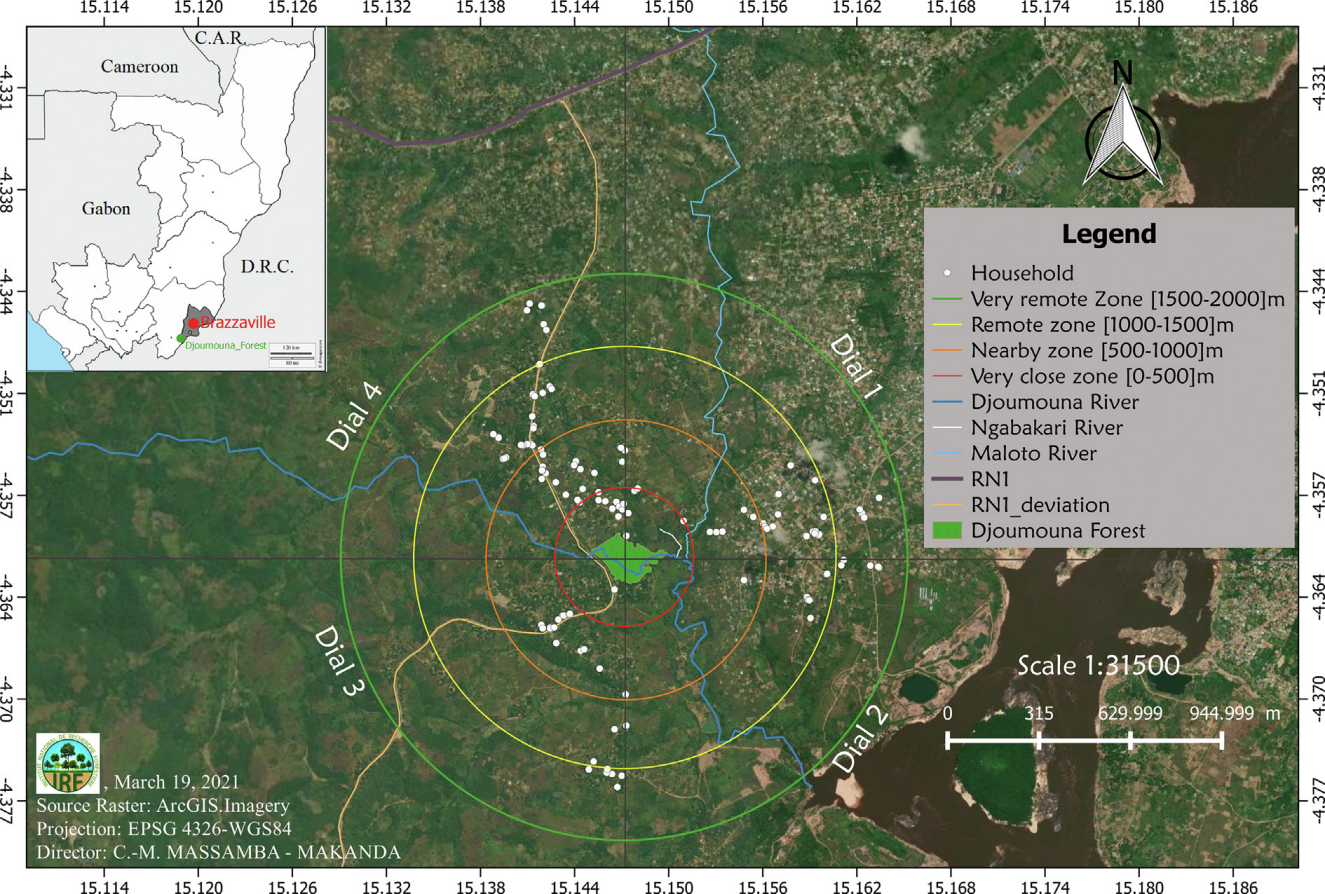
Experimental device and geo-referenced location of survey participants interviewed for socio-economic and ethnobotanical surveys.

The data were collected in the supposed area of influence of the Djoumouna forest, which extends over a radius of 2 km around the said ecosystem (Figure 4). This area of influence was divided into four 500 m wide concentric bands called ‘zones,’ considering the forest as ground zero (0). Zone 1 was located between the forest and in a 500 m radius buffer area (very close sector); zone 2, 3, and 4 fell within radii of between 500 and 1000 m (close sector), 1000 and 1500 m (remote sector), and between 1500 and 2000 m (very remote sector), respectively. This zoning was subdivided into four dials with dials 1, 2, 3, and 4 covering the northeastern, southeastern, southwestern and northwestern parts, respectively (Dieng et al., 2016).

Surveys were carried out in 264 households making it possible to understand the ethnobotanical value of the Djoumouna peri-urban forest.

The questionnaire consisted of multiple-choice questions relating to the identification of tangible benefits of fruits, wood, and medicinal products derived from this forest area. The choice of households surveyed was made at random in the different zones of each dial. The geographical coordinates of the households surveyed were recorded in order to take into account the geographical position of local residents in the vicinity of the Djoumouna forest.

#### 2.3.2 Data on the group of survey participants

The selection of survey participants was based on free consent and two simple criteria: participants had (i) to know of and be able to locate Djoumouna forest; and (ii) to frequent and/or exploit its biological resources. On this voluntary basis, one person was interviewed per household and, depending on the composition of the household, this responsibility fell to the head of the household or even one of the children meeting the criteria. Concerning the criteria, the sample size was not a function of the number of inhabitants in the study area, but rather of the practices of those inhabitants. Among the 264 households targeted, almost ¾ (73%) agreed to take part in the exercise, 19% of which claimed to not be aware of the existence of Djoumouna Forest. Conversely, 27% refused to participate. Taking gender into account, the group of survey participants was divided almost equally.

For 143 households that took part in the survey, nearly 3/4 (72.8%) live in equal proportions in zones 2 and 3. Zones 1 and 4, however, were home to 11.2% and 16.1% of households, respectively. According to the geo-referencing of households, three main axes existed: East (Dial 1), Southwest (Dial 3), and Northwest (Dial 4).

The participants subjects surveyed, whose ages ranged between 15 to over 45 years, belonged to several social strata. The specification of survey participants by age group revealed four age brackets spanning ten years each (Table 1). The majority of the survey participants had a secondary school education (56%), followed by primary school (31.5%) and university (2.8%). Finally, the unschooled fringe completed the remaining 9.8%.

**Table 1:** Data related to the group of survey participants.

Gender and age groups	Zone 1	Zone 2	Zone 3	Zone 4	Total
Men	8	24	27	13	72
Women	8	28	25	10	71
15–25 years	5	16	12	7	40
25–35 years	2	9	9	0	20
35–45 years	3	6	9	6	24
45 years and over	6	21	22	10	59
Total	16	52	52	23	143

An analysis of marital status for both genders combined revealed that 54.6% of survey participants were married; 42% were single and 3.5% were divorced. The analysis of socio-professional categories showed that of the group of survey participants, more than ¾ were workers and the remaining portion was made up of students (11.2%), civil servants (6.3%), the unemployed (2.8%) and the retired (2.1%).

#### 2.3.3 Data processing

The data collected were analyzed based on some ethnobotanical indicators, such as: frequency of citation (FC); use value (UV), informant consensus factor (ICF), and the level of fidelity (LF).

##### 2.3.3.1 Frequency of citation

Citation frequency (CF) was calculated for each ecosystem service category, as shown in the works of Sarr et al. (2013), Ngom et al. (2014) and Dieng et al. (2016).

(1) CF = (Number of citations / number of respondents) ×100

##### 2.3.3.2 Use value

For each category of ecosystem services, a Use Value (UV) as defined by Phillips et al. (1994) was calculated. The use-value provides a way of expressing the relative importance of ecosystem services in riparian areas (Ayantunde et al., 2009; Dossou et al., 2012; Sop et al., 2012; Dieng et al., 2016; Kimpouni et al., 2019):

(2) $$UV=\frac{\sum_{i}^{n}Ui}{\text{n}}\;\text{either}\;\text{UVt}=\overset{p}{\underset{1}{\sum }}\text{UV}$$

where *U*_i_ = number of citations for each ecosystem service and *n* = total number of survey participants.

##### 2.3.3.3 Informant consensus factor

The Informant Consensus Factor (ICF) as defined by Heinrich et al. (1998) is generally used in the field of ethno-medicine to identify culturally important species, determine their uses and hypothetically consider their further study (Heinrich et al., 1998; Andrade-Cetto and Heinrich, 2011; Uddin and Hassan, 2014; Kimpouni et al., 2019); in this paper, we have used the term “survey participant” to mean “informant.” The value of the ICF varies between 0 and 1 and indicates a high consensus when it tends towards 1. In this study, the ICF was calculated for each of the three categories of ecosystem services:

(3) ICF = (Nur – N1) / (Nur – 1)

where Nur = the number of citations in each ecosystem service category and N1 = the number of ecosystem services that comprise it.

##### 2.3.3.4 Level of fidelity

This indicator made it possible to calculate the level of fidelity (LF) of survey participants to the ecosystem services offered by the Djoumouna forest. The LF was calculated using Eq. (4) as defined by Friedman et al. (1986):

(4) LF = (Np / N) x 100

where Np = number of people who cited a type of ecosystem service or use and *N* = total number of people who derive some ecosystem service from that type of service.

## 3 Results

### 3.1 Biodiversity data

The inventory of biodiversity used by survey participants can be divided into the two major components of biodiversity (flora and fauna) with 13 species and 14 taxa used by the participants, respectively (Tables 2 and 3). Most of the species inventoried are used in traditional food and phytotherapy. Knowing that the Djoumouna peri-urban forest is surrounded by savannah, several species specific to this ecosystem and to fallow land were taken into account. This was true in the case of plants such as *Senna occidentalis* and *Sarcocephalus latifolius*, for various mushrooms and certain animal species such as *Brachytrupes membranaceus* (crickets) and *Thryonomys swinderianus* (aulacodes) that are dependent on crops and fallow land or dependent even on savannahs, as well as species that are dependent on the human environment (*Civettictis civetta* and *Nandinia binotata*). The two groups of Non-Ligneous Forest Products (NTFPs) had very high values for all parameters.

**Table 2:** Synthesis on the main categories of Non-Timber Forest Products (NTFPs).

Types and origins of NTFPs	N_ur_	Citation rate (%)	Nt	ICF
Plant	120	56,07	13	0,89
Animal	94	43,93	14	0,86
Total	214	100	27	1,75

Nur, number of citations in each type of NTFP; Nt, number of NTFPs in each category; ICF, Informant Consensus Factor.

**Table 3:** Data on ethnobotanical values of Non-Timber Forest Products (NTFPs) based on the distance from the forest.

NTFPs			Zone UV				Zone FC (%)				Zone LF (%)			
			1	2	3	4	1	2	3	4	1	2	3	4
Plant origin	*Quassia africana* (Baill.) Baill.	Simaroubaceae	0,18	0,06	0,08	0,09	18,75	5,77	7,69	8,70	11,11	5,88	11,76	25
	*Sarcocephalus latifolius* (Sm.) E.A.Bruce	Rubiaceae	0,18	0,02	0,04	0,04	18,75	1,92	3,85	4,35	11,11	1,96	5,88	12,5
	*Mushrooms (various species)*	-	0,56	0,31	0,19	0,09	50	30,76	19,23	8,70	29,63	31,37	29,41	25
	*Fruits (various species)*	-	0,31	0,35	0,15	0,13	31,25	34,62	15,38	13,04	18,52	35,29	23,52	37,5
	*Laccosperma secundiflorum* (P. Beauv.) Kuntze	Arecaceae	0,19	0,15	0,08	0	18,75	15,38	7,69	0	11,11	15,69	11,76	0
	*Gnetum africanum* Welw.	Gnetaceae	0,31	0,04	0,04	0	31,25	3,85	3,85	0	18,52	3,92	5,88	0
	*Aframomum stipulatum* (Gagnep.) K.Schum.	Zingiberaceae	0	0	0,02	0	0	0	1,92	0	0	0	2,94	0
	*Heisteria parvifolia* Sm.	Olacaceae	0	0,02	0	0	0	1,92	0	0	0	1,96	0	0
	*Hua gabonii* Pierre ex De Wild.	Huaceae	0	0,02	0	0	0	1,92	0	0	0	1,96	0	0
	*Millettia eetveldeana* (Micheli) Hauman	Fabaceae	0	0	0,02	0	0	0	1,92	0	0	0	2,94	0
	*Ongokea gore* (Hua) Pierre	Olacaceae	0	0	0,02	0	0	0	1,92	0	0	0	2,94	0
	*Senna occidentalis* (L.)Link.	Fabaceae	0	0	0,02	0	0	0	1,92	0	0	0	2,94	0
	*Sclerocroton cornutus* (Pax)Kruijt & Roebers	Euphorbiaceae	0	0,02	0	0	0	1,92	0	0	0	1,96	0	0
Animal origin	*Thryonomys swinderianus* Temminck	Thryonomyidae	0	0,02	0	0,04	0	1,92	0	4,35	0	1,96	0	12,5
	*Nandinia binotata* Gray	Viverridae	0	0,02	0	0	0	1,92	0	0	0	1,96	0	0
	*Varanus niloticus* Linnaeus	Varanidae	0	0	0,02	0	0	0	1,92	0	0	0	2,94	0
	*Tragelaphus spekii* J.H.Speke	Bovidae	0	0,02	0	0	0	1,92	0	0	0	1,96	0	0
	*Perodicticus potto* Müller	Lorisidae	0	0,02	0	0	0	1,92	0	0	0	1,96	0	0
	*Civettictis civetta* Schreber	Viverridae	0	0,02	0	0	0	1,92	0	0	0	1,96	0	0
	*Fish (various species)*	-	0,5	0,19	0,19	0,04	50	19,23	19,23	4,35	29,63	19,60	29,41	12,5
	*Rats (various species)*	-	0,38	0,12	0,08	0,04	31,25	11,54	7,69	4,35	18,51	11,76	11,76	12,5
	*Caterpillar (various species)*	-	0,19	0,08	0,08	0	18,75	7,69	7,69	0	11,11	7,84	11,76	0
	*Birds (various species)*	-	0,25	0,06	0,06	0	25	5,77	5,77	0	14,81	5,88	8,82	0
	*Brachytrupes membranaceus* Drury	Gryllidae	0,06	0,04	0,04	0	0,06	3,85	3,85	0	3,70	3,92	5,88	0
	*Fruit bats (various species)*	-	0,13	0,04	0	0	12,5	3,85	0	0	7,40	3,92	0	0
	*Squirrels (various species)*	-	0,19	0	0	0	18,75	0	0	0	11,11	0	0	0
	*Snakes (various species)*	-	0,25	0,08	0,08	0,04	25	7,69	7,69	4,35	14,81	7,84	11,76	12,5

### 3.2 Ethnobotanical value of plant-based NTFPs

#### 3.2.1 Frequency of citation of NTFPs

**Figure 5:**
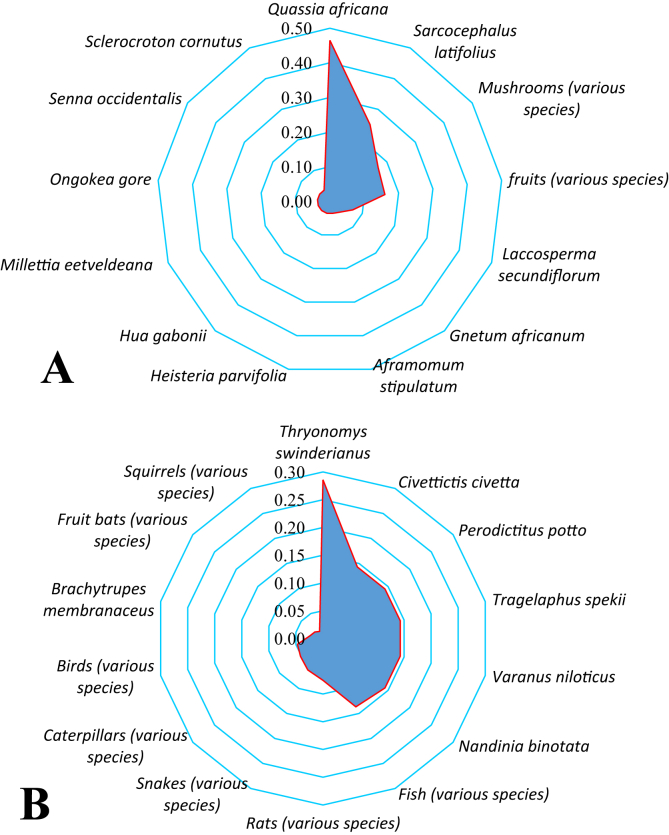
Frequency of citation of Non-Timber Forest Products (NTFPs) associated with the Djoumouna peri-urban forest. (A) plant or (B) animal origin.

The citation frequency for all taxa was less than 50% meaning fewer than 50% of all survey participants discussed using any one item. With citation frequency values ranging from 3.60 to 46.50%, more than 69% of the taxa surveyed had a citation frequency of less than 10% (Figure 5A). The majority of the remainder had a citation frequency in the range 16.03–25%, although that of *Quassia africana* was 46.5%. The taxa with the highest frequency were associated with phytotherapy (*Sarcocephalus latifolius, Ongokea gore* and *Quassia africana*) and food (various fruits and mushrooms).

As for NTFPs of animal origin, citation frequencies were very low; the highest were associated with herbivores that consumed crops (*Thryonomys swinderianus*) and avian peasants (*Nandinia binotata*, *Civettictis civetta* and *Varanus niloticus*), with exceptions of two mostly herbivorous mammals, *Tragelaphus spekii* and *Perodicticus potto.* The low citation frequency rates (≤15%) reveal that the Djoumouna peri-urban forest has very small wildlife populations (Figure 5B).

### 3.3 Ethnobotanical data by area

#### 3.3.1 Use values of different NTFPs

Ethnobotanical data values reveal that less than 50% of the NTFPs appearing in survey results were unanimously reported in all four zones. For example, 15.38% of NTFPs were not cited in zone 4, while 53.85% of NTFPs were exploited in only one of the four zones (Table 3). In addition to the products that are known from only one zone, the ethnobotanical use-value decreases with increasing distance from the Forest.

As with NTFPs of plant origin, the trend observed is also true for those of animal origin. The ethnobotanical use-value decreased with distance with increasing distance from the Forest. Since wildlife is rarely observed in and around this ecosystem, especially “big” game such as *Thryonomys swinderianus*, *Nandinia binotata*, *Civettictis civetta*, *Varanus niloticus*, *Tragelaphus spekii* and *Perodicticus potto*, the ethnobotanical use value revealed various exploitation differences between areas. The fauna category that was not inventoried in Zone 1 was reported almost exclusively in Zone 2 (Table 3).

Informant consensus factors for both NTFP categories were very high. The values for each were 0.89 for plant and 0.86 for wildlife NTFPs. In relation to the four study areas, the values of this parameter ranged from 0.57 to 0.84 for plant NTFPs and 0 to 0.82 for wildlife species. From zone 1 to zone 4, the values of the survey participant consensus factor decreased with increasing distance from the Forest regardless of the origin of the NTFPs considered.

#### 3.3.2 Citation frequency

The general trend for the frequencies of reports of use of various NTFPs was a decrease frequency intimately associated with an increased distance from the forest, although there were a few rare cases where there was a slight increase in the rate of citation with increased distance from the forest. However, the data on the level of fidelity show fluctuations depending on the area under study. This parameter, which is independent of remoteness, would be a function of the daily needs of local residents, such as needs for food and traditional herbal medicine. These observations apply to both groups floral and faunal NTFP species (Table 3).

#### 3.3.3 Fidelity level

Despite some minor fluctuations between areas, fidelity values generally decreased with increasing distance (Table 3). The popular products are those that are used daily in traditional pharmacopoeia and food. It should be noted that the level of fidelity would be a function of empirical knowledge of the virtues of plants and animal captured, or even maximum exploitation of the surrounding ecosystems.

### 3.4 Data on ethnobotanical values according to age groups

#### 3.4.1 Frequency of citation of each NTFP by age group

The frequencies of citations was higher in the interval formed by the age groups of 15–45; beyond that, the values of this parameter dropped sharply. The 45 + age group had five to more than ten times fewer citations than in the 25–35 age group (Table 4). In majority of the cases, the best citation performances were noted in the 25–35 age group. Once again, taxa associated with food and popular traditional herbal medicine were highlighted.

**Table 4:** Data on ethnobotanical values of NTFPs by age range.

NTFPs			Frequency Citation (%)				Ethnobotanical Use Value (%)				Level Fidelity (%)			
			15–25 years	25–35 years	35–45 years	45 years and over	15–25 years	25–35 years	35–45 years	45 years and over	15–25 years	25–35 years	35–45 years	45 years and over
Plant origin	*Quassia africana* (Baill.) Baill.	Simaroubaceae	7,5	15	16,67	3,39	0,15	0,05	0,04	0,07	12,77	0,07	16,67	7,55
	*Sarcocephalus latifolius* (Sm.) E.A.Bruce	Rubiaceae	7,5	5	8,33	1,69	0,1	0	0	0,05	8,51	0	0	5,66
	*Mushrooms (various species)*	-	20	80	41,67	3,39	0,33	0,2	0,04	0,31	27,66	28,57	16,67	33,96
	*Fruits (various species)*	-	12,5	90	33,33	5,08	0,33	0,2	0,13	0,24	27,66	28,57	50	26,42
	*Laccosperma secundiflorum* (P. Beauv.) Kuntze	Arecaceae	7,5	40	16,67	0	0,15	0,15	0	0,10	12,77	21,43	0	11,32
	*Gnetum africanum* Welw.	Gnetaceae	12,5	10	8,33	0	0,05	0,1	0,04	0,07	4,26	14,29	16,67	7,55
	*Aframomum stipulatum* (Gagnep.) K.Schum.	Zingiberaceae	0	0	4,17	0	0,03	0	0	0	2,13	0	0	0
	*Heisteria parvifolia* Sm.	Olacaceae	0	5	0	0	0	0	0	0,02	0	0	0	1,89
	*Hua gabonii* Pierre ex De Wild.	Huaceae	0	5	0	0	0	0	0	0,02	0	0	0	1,89
	*Millettia eetveldeana* (Micheli) Hauman	Fabaceae	0	0	4,17	0	0,03	0	0	0	2,13	0	0	0
	*Ongokea gore* (Hua) Pierre	Olacaceae	0	0	4,17	0	0,03	0	0	0	2,13	0	0	0
	*Senna occidentalis* (L.) Link.	Fabaceae	0	0	4,17	0	0	0	0	0,02	0	0	0	1,89
	*Sclerocroton cornutus* (Pax)Kruijt & Roebers	Euphorbiaceae	0	5	0	0	0	0	0	0,02	0	0	0	1,89
Animal origin	*Thryonomys swinderianus* Temminck	Thryonomyidae	2,5	0	4,16	0	0,03	0	0,04	0	2,5	0	20	0
	*Nandinia binotata* Gray	Viverridae	0	0	0	1,69	0	0	0	0,02	0	0	0	2,63
	*Varanus niloticus* Linnaeus	Varanidae	0	5	0	0	0	0,05	0	0	0	9,09	0	0
	*Tragelaphus spekii* J.H.Speke	Bovidae	0	0	0	1,69	0	0	0	0,02	0	0	0	2,63
	*Perodicticus potto* Müller	Lorisidae	2,5	0	0	0	0,03	0	0	0	2,5	0	0	0
	*Civettictis civetta* Schreber	Viverridae	0	0	0	1,69	0	0	0	0,02	0	0	0	2,63
	*Fish (various species)*	-	32,5	15	4,16	20,34	0,33	0,15	0,04	0,20	32,5	27,27	20	31,58
	*Rats (various species)*	-	15	10	4,16	11,86	0,15	0,1	0,04	0,12	15	18,18	20	18,42
	*Caterpillars (various species)*	-	7,5	10	4,16	8,47	0,08	0,1	0,04	0,08	7,5	18,18	20	13,16
	*Birds (various species)*	-	12,5	5	0	6,78	0,13	0,05	0	0,07	12,5	9,09	0	10,53
	*Brachytrupes membranaceus* Drury	Gryllidae	10	0	0	1,69	0,1	0	0	0,02	10	0	0	2,63
	*Fruit bats (various. species)*	-	5	0	0	3,39	0,05	0	0	0,03	5	0	0	5,26
	*Squirrels (various species)*	-	0	0	4,16	3,39	0	0	0,04	0,03	0	0	20	5,26
	*Snakes (various species)*	-	12,5	10	0	3,39	0,13	0,1	0	0,03	12,5	18,18	0	5,26

In relation to fauna, citation frequencies were very low and varied from 1.69 to 32.5% for all age groups. The fauna being very poorly represented, the highest frequency was associated with fish and the lowest with “big” game species. The age group of 45 years and older appeared to be the age group that benefits most from the big game products of this ecosystem (Table 4).

#### 3.4.2 Ethnobotanical use values

The ethnobotanical use value by age group revealed a strong disparity among the actors (Table 4). The age groups of 15–25 years and 45 years and older were the most involved in NTFP harvesting, with particular emphasis on the first age category for plant-based NTFPs. With experience in tracking animals being an asset, those 45 years of age and older are better prepared for wildlife harvesting. Differences observed could be a result of social conditions characterizing each survey participant group.

#### 3.4.3 Informant consensus factor

Informant Consensus Factor data by age ranged from 0.5 to 0.78 for plant-based NTFPs and from 0 to 0.79 for animal-based NTFPs. In relation to age ranges, the value of the survey participant consensus factor was almost the same for 15–25-year-olds (0.78–0.79) and those 45 years and older (0.73–0.76) regardless of the type of NTFP. In contrast, for those aged 25–35 and 35–45, ICF had notable differences between plant and animal NTFPs. For both age groups, this value was 0.67 and 0.5 for the plant type and 0.5 and 0 for the wildlife type.

#### 3.4.4 Data on the level of fidelity of use of NTFPs

The values of the level of fidelity of use of NTFP by age group show that this parameter increased almost correlatively with age in the first three classes (Table 4). Despite some variation within the age groups, the majority of loyalty values were below 50%. As with all other parameters, the needs expressed do not cover all age groups, and the best provided were for those aged 15–25 and 45 and older.

#### 3.4.5 Gender data and ethnobotanical use value

Regardless of the parameter and/or taxon mentioned, men had an exploitation rate of 100%, while that of women was well below 50% (Table 5). A close analysis of the values revealed that women had very low ethnobotanical use values no matter which parameter was considered. The reason for these values may lie in the social structure of the group of survey participants and in the difference in the possession of knowledge between genders.

**Table 5:** Data on ethnobotanical use values of Non-Timber Forest Products (NTFPs) by gender of interviewee.

NTFPs			Use Value		Frequency Citation (%)		Level Fidelity (%)	
			Men	Women	Men	Women	Men	Women
Plant origin	*Quassia africana* (Baill.)Baill.	Simaroubaceae	0,03	0	2,77	0	2,5	0
	*Sarcocephalus latifolius* (Sm.) E.A.Bruce	Rubiaceae	0,01	0	1,39	0	1,25	0
	*Mushrooms (various species)*	-	0,01	0	1,39	0	1,25	0
	*Fruits (various species)*	-	0,01	0	1,39	0	1,25	0
	*Laccosperma secundiflorum* (P.Beauv.) Kuntze	Arecaceae	0,01	0	1,39	0	1,25	0
	*Gnetum africanum* Welw.	Gnetaceae	0,01	0	1,39	0	1,25	0
	*Aframomum stipulatum* (Gagnep.) K.Schum.	Zingiberaceae	0,33	0,07	33,33	7,04	30	12,5
	*Heisteria parvifolia* Sm.	Olacaceae	0,19	0,03	19,44	2,82	17,5	5
	*Hua gabonii* Pierre ex De Wild.	Huaceae	0,14	0,01	13,89	1,41	12,5	2,5
	*Millettia eetveldeana* (Micheli) Hauman	Fabaceae	0,11	0,03	11,11	2,82	10	5
	*Ongokea gore* (Hua) Pierre	Olacaceae	0,04	0,03	4,17	2,82	3,75	5
	*Senna occidentalis* (L.) Link.	Fabaceae	0,04	0,01	4,17	1,41	3,75	2,5
	*Sclerocroton cornutus* (Pax) Kruijt & Roebers	Euphorbiaceae	0,04	0	4,17	0	3,75	0
Animal origin	*Thryonomys swinderianus* Temminck	Thryonomyidae	0,03	0	2,78	0	2,5	0
	*Nandinia binotata* Gray	Viverridae	0,01	0	1,39	0	1,25	0
	*Varanus niloticus* Linnaeus	Varanidae	0,01	0	1,39	0	1,25	0
	*Tragelaphus spekii* J.H. Speke	Bovidae	0,01	0	1,39	0	1,25	0
	*Perodicticus potto* Müller	Lorisidae	0,01	0	1,39	0	1,25	0
	*Civettictis civetta* Schreber	Viverridae	0,01	0	1,39	0	1,25	0
	*Fish (various species)*	-	0,33	0,07	33,33	7,04	30	35,71
	*Rats (various species)*	-	0,19	0,03	19,44	2,82	17,5	14,29
	*Caterpillars (various species)*	-	0,14	0,01	13,89	1,41	12,5	7,14
	*Birds (various species)*	-	0,11	0,03	11,11	2,82	10	14,29
	*Brachytrupes membranaceus* Drury	Gryllidae	0,04	0,03	4,17	2,82	3,75	14,29
	*Fruit bats (various species)*	-	0,04	0,01	4,17	1,41	3,75	7,14
	*Squirrels (various species)*	-	0,04	0	4,17	0	3,75	0
	*Snakes (various species)*	-	0,11	0,01	11,11	1,41	10	7,14

#### 3.4.6 Informant consensus factor by gender

The values of the survey participant consensus factor by gender ranged from 0.54 to 0.86. For males, the values were 0.86 for plant-based NTFPs and 0.84 for wildlife. However, for females, the values were 0.85 for plant-based NTFPs and 0.54 for animal-based NTFPs.

### 3.5 Medicinal plants discussed by the residents of the Djoumouna forest

In the field of traditional pharmacopoeia, in addition to plants from the surrounding savannah such as *Sarcocephalus latifolius*, *Aframomum stipulatum* and *Senna occidentalis*, the Djoumouna forest has a significant phytotherapeutic potential for the local population (Table 6). In fact, two-thirds of the phytotherapeutic base of the local residents is derived from the forest, compared to one-third from the surrounding savannah. Despite the fact that *Sarcocephalus latifolius* and *Quassia africana* are associated with the prevalence of malaria, the ethnobotany component revealed that high citation rates were associated with multiple-use plants such as *Aframomum stipulatum*, *Heisteria parvifolia* and *Hua gabonii*. The gender exploitation rates of NTFPs indicate a dominance of males, hence a level of traditional knowledge in their favor. Finally, the approach to the exploitation of these plants according to age groups shows *Quassia africana* and *Sarcocephalus latifolius* were primarily used.

**Table 6:** Diseases treated by specific medicinal plants.

Taxa	Family	Local Names (laari)	Morphological type	Part used	Condition treated
*Quassia africana* (Baill.) Baill.	Simaroubaceae	Mumpessi	Shrub	Roots	Malaria
*Sarcocephalus latifolius* (Sm.) E.A.Bruce	Rubiaceae	Tsiénga	Shrub	Roots	Malaria
*Aframomum stipulatum* (Gagnep.) K.Schum.	Zingiberaceae	Ntundu	Perennial Grass	Fruits	General Asthenia
*Heisteria parvifolia* Sm.	Olacaceae	Ndolo	Tree	Leaves Bark	Asthma, Menstrual disorder
*Ongokea gore* (Hua) Pierre		Muwumi wa sangui	Tree	Bark	Constipation
*Hua gabonii* Pierre ex De Wild.	Huaceae	Mumpimpiti	Tree	Bark	Medico- magical
*Millettia eetveldeana* (Micheli) Hauman	Fabaceae	Mubuéngué	Tree	Sheets	Constipation
*Senna occidentalis* (L.) Link.	Fabaceae	Kinkéliba	Shrub	Root	Sexual Asthenia
*Sclerocroton cornutus* (Pax) Kruijt & Roebers	Euphorbiaceae	Ntiti	Shrub	Sheets	Yellow Fever

### 3.6 Wild animals hunted by the residents of the Djoumouna forest

In the area of wildlife, the animals hunted in the Djoumouna forest and its surroundings are mostly associated with the presence of humans, particularly their activities (crops and bird breeding). These faunal species represent a little more than two-thirds of the total hunted species in this peri-urban forest (Figure 5). Within the framework of this research, the local populations cited six species that belong to five families and six genera (Tables 5 and 7).

**Table 7:** Wild animals hunted in the Djoumouna forest.

Taxa	Family	Local name	
		commun	laari
*Varanus niloticus* Linneaus	Varanidae	Varan	Mbambi
*Perodicticus potto* Müller	Lorisidae	*-*	Ntsikanda
*Thryonomys swinderianus* Temminck	Thryonomyidae	Aulacode	Ntsibizi
*Nandinia binotata* Gray	Viverridae	Nandine	Mbala
*Civettictis civetta* Schreber		Civette	Nzobo
*Tragelaphus spekii* J.H.Speke	Bovidae	Sitatunga	Nkabi

## 4 Discussion

### 4.1 Biodiversity analysis

The peri-urban formation of the Djoumouna based on its phytogeographic position is an element of the dense tropical rainforest (Kimpouni et al., 2017, 2018a, 2018b). Rich in species, these ecosystems support the socio-cultural base of traditional communities, as noted in the works of Bouquet (1969), Adjanohoun et al. (1988), Kimpouni and Motom (2012), and Kimpouni et al. (2018a, 2018b, 2019). In relation to the known ethnobotanical value of the Congolese forests, the Djoumouna peri-urban forest is a very poor facies. This parameter which combines floristic diversity and traditional pharmacopoeia is a corollary of its very advanced state of degradation and/or the low level of empirical knowledge of the riparian populations of the ecosystem services provided by the plants (Kimpouni et al., 2017, 2018a, 2018b). These observations are supported by the failure of survey participants to cite several important species of traditional herbal medicine and handicrafts such as *Pausinystalia yohimbe*, *Staudtia kamerunensis*, *Phylocosmus africanus*, and *Canarium schweinfurthii*, which were present but with most individuals bearing the marks of organ removal.

As for wildlife diversity, it is interesting to note that the level of degradation and the frequency of human visitation of this peri-urban forest have resulted in this forest not being able to support a representative and significant animal load, despite its status as a private forest. Data from ethnobotanical and/or ethnozoological indicators are a sure indicator of the accidental presence of these animals in this forest, except for taxa that are partly dependent on human activities such as predators including *Nandinia binotata* and *Civettictis civetta* for the avian fauna of peasant farming as well as *Thryonomys swinderianus* and *Tragelaphus spekii* for the peasant crops of *Manihot esculenta*, *Zea mays*, and *Arachis hypogaea*. Monitoring of this flora could provide information on several aspects of their presence and role in this forest formation.

### 4.2 Ethnosociological analysis

The geographical location of the Djoumouna peri-urban forest and the socio-professional situation and/or even the level of education of the group of survey participants may also be factors that have strongly influenced the data collected. Indeed, the low rates of survey participants who were not in school and were unemployed could explain the low values of the ethnobotanical and/or ethnozoological indicators. However, the majority of survey participants were said to be endowed with traditional knowledge on the virtues of plants, or even animals, without materializing these acquired or even innate values. This observation would be supported by the fact that within each of us lies the intrinsic foundations of the socio-cultural base of community traditions (Kimpouni et al., 2017, 2018a, 2018b, 2019a, 2019b). Far from acculturation, the residents of the Djoumouna peri-urban forest have greater recourse to modern medicine than many people. This practice is in line with the level of education of the survey participants and the secondary acquisitions resulting from the mixing of customs (Kimpouni and Motom, 2012). Despite the proven therapeutic power of the plants surveyed, very few of the people surveyed put the values of this cultural background into practice. It should be noted that throughout the world and particularly in Africa, about 80% of the population use traditional phytotherapy to cover primary health care needs (WHO, 2002; Wezel, 2002; Kimpouni et al., 2017, 2018a, 2018b, 2019a, 2019b).

The use of the pharmacognosic properties of plants reflects an empirical and experimental acquisition of knowledge related to those properties. These benefits, which are the foundation of the socio-cultural base of traditional societies, are transmitted orally from generation to generation (Badiaga, 2011; Kimpouni et al., 2017, 2019a). The observation of ethnobotanical data in relation to age groups confirms the gradual acquisition of knowledge. However, this same data also shows that the low level of interest among young people in resorting to traditional medicine is a proven threat to the sustainability of this knowledge (Kimpouni et al., 2018a, 2018b, 2019a). This imprint is more marked in urban and suburban areas where the mundane seems to be the rule of existence (Kimpouni et al., 2017, 2019b).

### 4.3 Analysis of the level of ethnobotanical exploitation by gender

The ethnobotanical and ethnozoological data collected in the present study provide information on speciation of NTFP activities and exploitation of resources within genders. However, the scarcity and level of anthropogenic pressure on NTFPs do not allow a proper assessment of this specialization. Nevertheless, hunting and gathering activities are said to be male functions, compared to almost nothing along those lines for women. The values of the ethnobotanical indicators confirm this observation and seem to offer a good share of the frequentation of the Djoumouna peri-urban forest to men. Since the survey was conducted in a peri-urban environment and given the social composition of the group of survey participants, the combination of these two factors can explain the male hegemony over the NTFPs in this ecosystem. This result seems to be the opposite of the rural world where the woman, generally the guarantor of empirical knowledge, are responsible for the education of children, without denying the function of women as providers of food NTFPs to the household (Wezel, 2002; Kimpouni et al., 2017, 2018a, 2018b, 2019b).

### 4.4 Ethnobotanical value analysis

The taxa collected from the Djoumouna peri-urban forest represent a reliable source of medicines and proteins, both animal and plant, for the local people who are closely dependent on them. Finally, others on the social scale find a significant source of income through the cyclical and/or occasional sale of these NTFPs (Ingram et al., 2016; Tabuna, 2016).

The plants inventoried during this survey have proven medicinal virtues and are used for various ailments and uses by traditional communities throughout their geographical area (Nsonde Ntandou et al., 2020). The majority of them have also been subjected to phytochemical analyses that have confirmed their therapeutic powers (Nsonde Ntandou et al., 2020). Examples include taxa such as *Quassia africana, Ongokea gore, Sarcocephalus latifolius, Senna occidentalis* and *Sclerocroton cornutus*, whose pharmacognosic composition has been unequivocally established.

## 5 Conclusion

The investigations carried out within the framework of this study of humans and their environment highlighted the ethnobotanical and sociological values associated with peri-urban forests. At the same time, the survey revealed that in peri-urban or even urban environments, the effects of the modern world are eroding empirical knowledge which provides the foundation of the socio-cultural base of any traditional community. This loss of value may be irreversible insofar as (i) the vestiges of the knowledge in possession remain theoretical and never materialized, and (ii) the shrinkage of the younger generations that have this knowledge and know its benefits which creates obstacles to the perpetuation of the culture. This view is supported by the difference in the level of knowledge between age groups, which constitutes a threat to the said traditional knowledge.

With the Djoumouna peri-urban forest, the inventory data reveal a highly anthropized ecosystem that provides biodiversity capable of ensuring the primary needs of local residents. For these reasons, another look, or even a special emphasis, should be given to this ecosystem in order to safeguard this biodiversity that is being sacrificed while disregarding its proven value.

## Declarations

### Author contribution statement

Kimpouni V., Nzila J.D.D.: Conceived and designed the experiments; Analyzed and interpreted the data; Wrote the paper.

Watha-Ndoudy N.: Performed the experiments; Analyzed and interpreted the data; Wrote the paper.

Madzella-Mbiemo M.I.: Performed the experiments; Contributed reagents, materials, analysis tools or data; Wrote the paper.

Yallo Mouhamed S.: Performed the experiments; Contributed reagents, materials, analysis tools or data.

Kampe J.-P: Performed the experiments; Contributed reagents, materials, analysis tools or data.

### Funding statement

This research did not receive any specific grant from funding agencies in the public, commercial, or not-for-profit sectors.

### Data availability statement

Data included in article/supplementary material/referenced in article.

### Declaration of interests statement

The authors declare no conflict of interest.

### Additional information

No additional information is available for this paper.
